# Adolescents’ exposure to and evaluation of food promotions on social media: a multi-method approach

**DOI:** 10.1186/s12966-022-01310-3

**Published:** 2022-06-27

**Authors:** D. L. M. van der Bend, T Jakstas, E van Kleef, V. A. Shrewsbury, T Bucher

**Affiliations:** 1 School of Health Sciences, College of Health, Medicine and Wellbeing, The University of Newcastle, Callaghan NSW, University Drive, Newcastle, 2308 Australia; 2 Marketing and Consumer Behaviour Group, Department of Social Sciences, Wageningen University & Research, Hollandseweg 1, 6706 KN Wageningen, the Netherlands; 3 School of Environmental and Life Sciences, College of Engineering, Science and Environment, The University of Newcastle, 10 Chittaway Rd, Ourimbah, NSW 2258 Australia

**Keywords:** Social media, Food, Marketing, Promotions, Adolescents

## Abstract

**Background:**

Traditional food marketing, mostly involving advertisement of nutrient poor and energy dense foods, has the effect of enhancing attitudes, preferences, and increasing intake of marketed foods in adolescents, with detrimental consequences for health. While the use of social media applications in adolescents has proliferated, little is known about the content of food promotions within these applications. The aim of this study was to investigate adolescents’ exposure to and evaluation of social media food promotions (SMFPs).

**Methods:**

Australian adolescents aged 13–16 years joined one-on-one Zoom meetings with the researcher on the device they normally used for social media. Participants shared their screen and visited up to three of their favourite social media platforms for 10 min each, during which the researcher pointed out examples of SMFPs to participants. Next, participants answered questions about their awareness and appreciation of SMFPs. Screenshots of SMFPs were de-identified and analysed.

**Results:**

The study included 35 adolescents aged 14.4 (± 1.2) years (boys: *n* = 18; girls: *n* = 17). Instagram, Snapchat and YouTube were the most favoured social media platforms. During a total of 1000 min of viewing time, 1801 unbranded (*n* = 1221) and branded (*n* = 580) SMFPs were identified. Participants viewed a median rate (IQR) of 12.0 (6.3–20) SMFPs per 10 min, with a median rate of 6.0 (3–11) non-core SMFPs per 10 min. A majority of SMFPs (62%) were embedded into celebrity influencer or entertaining content (e.g., vlogs, cooking videos, streamed TV content). In total, 60% of the participants said they had sometimes, rarely or never noticed the SMFPs pointed out by the researcher themselves. Participants largely remembered non-core foods or brands (77%). Almost half (49%) of participants liked SMFPs, while only 6% disliked them.

**Conclusions:**

This study contributes to a relatively unexplored research area. The outcomes show adolescents’ SMFP exposure mostly concerns unhealthy foods, shown in advertisements and other food-related posts, which are integrated into a wide variety of entertainment that is appreciated by adolescents. The results emphasise the need for more research on SMFPs, with particular focus on the impact on adolescent dietary behaviours, and clearer definitions and stricter regulations regarding adolescent-targeted social media food marketing.

**Supplementary Information:**

The online version contains supplementary material available at 10.1186/s12966-022-01310-3.

## Introduction

Adolescents are among the most frequent consumers of snacks and fast-foods, and it has been noted across the literature that, as a group, they tend to skip meals or eat away from home [1]. These dietary behaviours are associated with an increased risk for weight gain and diet-related diseases such as type 2 diabetes or heart disease [1]. Food marketing of nutrient poor, energy dense foods, significantly contributes to adolescents’ consumption of these foods [2–4]. This impact of food promotions on adolescents’ consumption patterns can be explained by a cascade of effects, through which exposure to food promotions impacts adolescents’ cognitive stages (i.e., awareness) and affective stages (i.e., appreciation or liking), and hence unhealthy food purchases or consumption [5]. This is comparable to the hierarchy of effects (HoE) models that underpin social marketing effects [6] and that guide food marketers in predicting adolescents’ purchasing behaviour and encourage consumption of their brands [7].

In recent years, social media has become a particularly popular channel for marketers to promote foods or beverages to adolescents, as they are one of the most digitally-driven segments of the population. A 2018–2019 study found that a subset of Australian adolescents 13–17 years spent approximately 30 h per week on web-based media using mobile devices, desktop and laptop computers, and are most active on social media applications including Instagram, Facebook, Snapchat and music streaming apps [8]. However, trends in digital media are constantly changing, with new social media applications appearing and becoming popular among adolescents within a short period of time [9]. Compared to TV and print, social media offers food marketers a range of new possibilities to influence adolescents’ cognitive and affective stages and hence consumption. Namely, social media enables more implicit and personalised persuasion techniques that engage adolescents in emotional and entertaining experiences, encouraging them to share these experiences with their friends [10]. On social media, users can contribute to the food marketing process, resulting in either influencer- or user-generated marketing content, blurring the lines between online user activities and advertising [11, 12]. This leads to messages with high levels of food integration, with peers or influencers handling or consuming the product, which has been found to increase children’s attention to or choice of the product promoted [13].

While adolescents generally have a higher cognitive ability than younger children, it is suggested they are still vulnerable to highly integrated social media food marketing as they are in the key stage of identity development, making them want to conform to a certain image or peer group [11, 14]. Additionally, due to hormonal and neurological changes, adolescents may be more impulsive and likely to follow their peers’ risky behaviours instead of accepting guidance from parents or adults [10, 15]. For instance, adolescents were found to be more likely to believe their peers consume more non-core foods than core foods, suggesting that social media food messages may shape normative perceptions, and trigger unhealthy eating in adolescents [16]. Indeed, adolescents’ self-reported exposure to and engagement with non-core food marketing or food messages on social media have been positively associated with their self-reported intake of non-core foods [16, 17].

Social media food marketing (SMFM) affects adolescents globally because of its borderless nature, yet food marketing regulations are currently focused on children under 12 years and do not take into account the more overt and persuasive marketing techniques applied in digital media [18]. There has been no strict global mandatory regulations regarding adolescent-targeted SMFM to date. Therefore, continuous monitoring of the nature, extent and impact of food marketing to adolescents is warranted, as more complete evidence on all media marketing channels can inform further development and strengthening of regulations and policies [4, 19]. However, objective measurement of exposure to SMFM is practically difficult due to the dynamic and personalised nature of SMFM. Amongst others, ethical and privacy aspects now play a major role when wishing to access and investigate personal social media accounts, because informed consent cannot be obtained from all members or connections within the user’s network [20]. As a result, some studies on adolescent SMFM exposure used self-reported data based on questionnaires about frequency of exposure [16, 17]. Other exposure studies were focused on one platform and had methodological limitations such as restrictions to the type of screen recorded (i.e., no smartphone) or type of data collected (i.e., only advertising or a fixed list of brands investigated) [21, 22]. Others relied on self-collection of social media content from adolescents’ own social media accounts [8, 23]. To date, one study has conducted an objective measurement of adolescents’ full exposure to SMFM, showing the majority concerns fast food, sugar-sweetened beverages and snacks [24]. However, there have been no real-time full exposure studies that compared the presence and nature of branded food messages to unbranded food messages on social media, together referred to as social media food promotions (SMFPs) in this research. Also, objective measurements of adolescents’ individual exposure to SMFPs have not yet been linked directly to adolescents’ subjective interpretations of and experiences with SMFPs. In fact, qualitative studies on the impact of social media food marketing on diet-related outcomes in adolescents are highly limited [25]. The main objective of this study was to quantitatively investigate adolescents’ real-time exposure to SMFPs. To obtain additional contextual understanding of how adolescents perceive the SMFPs they are exposed to, their perceptions towards SMFPs were qualitatively explored, guided by key components of the HoE approach, i.e., awareness and appreciation.

## Methods

### Study design

This study was approved by the Human Research Ethics Committee of the University of Newcastle, Australia (Ref no: H-2019–0309). In total, three different data collection methods were combined to obtain both quantitative (i.e., survey and observation of SMFP exposure) and qualitative data (i.e., interviews). The survey was mainly used to check participant eligibility and to describe sample characteristics. The main focus was on the quantitative analysis of adolescents’ SMFP exposure. The subsequently collected qualitative interview data provided additional contextual understanding to the exposure data. Adding a qualitative component was deemed relevant as adolescents’ perceptions of SMFPs would give more in-depth insights into how they experienced their SMFP exposure. The below paragraphs elaborate on the data collection methods in more detail. Quantitative and qualitative data were analysed and elaborated on in separate results sections, and the sets of results were eventually integrated during the interpretation or discussion phase.

### Study setting and recruitment

Between October 2020 and June 2021, 13–16-year-olds were recruited to participate via researchers’ networks (word of mouth, email, social media), flyers, and other media channels including local radio stations and a local newspaper in the Hunter and Newcastle region. The only other eligibility criterion was that the adolescent had to be active on at least one social media platform at least once a week.

The study was completely online, including a questionnaire and interview via Zoom. Parents and participants who expressed interest were sent information sheets describing the study procedures and consent forms which they were both asked to sign before participation. After receiving the signed consent forms, adolescent participants were asked to fill out an online eligibility screening questionnaire on Qualtrics XM Platform. This questionnaire collected key demographic characteristics and data on participants’ social media use, their favourite social media platforms (i.e., up to three and at least one), and which device(s) participants normally used to access these platforms (Supplementary Table 1).

Next, participants were directed to a website to book a date and time for a one-on-one Zoom interview with the principal researcher. The principal researcher sent participants a link to an online Zoom meeting, asking them to access the link on the device they used most frequently to access their social media accounts. If participants used different devices for different social media accounts, they were asked to switch between devices during the meeting.

### Zoom session procedure

All Zoom sessions were held by the principal researcher, took up to 45 min and were screen-recorded. Zoom sessions commenced with the researcher introducing the study and describing the interview procedure. The researcher emphasised that the study purpose was to collect data on food content viewed by adolescents, not private data, and that this content would be de-identified and only anonymous data would be analysed. Parents were allowed to be present during this introduction, in case of questions and to meet the researcher. They were encouraged to leave after the introduction but could stay if this was the adolescent’s preference.

As described in the information sheet and verbally at the start of the interview, the researcher notified the participant that the screen recording had started, and participants were asked to use the share screen function within Zoom. Participants were then asked to open their favourite social media platforms, as indicated in the online questionnaire, one by one. This was either app- or web-based, depending on how they would normally access the platforms. Next, they were instructed to do what they would normally do for 10 min on each platform, e.g., scroll through their feeds, comment, share, like, click on links, etc. This procedure aimed to capture a snapshot of participants’ normal social media behaviour. The researcher monitored the time, and asked the participant to close each social media platform after 10 min. During each 10 min session, the principal researcher remained silent, except to point out examples of food content, food advertising, or food promotions to the participants if these appeared on screen.

After the scrolling activity, at the end of the Zoom session, adolescents were asked a series of questions to ascertain their appreciation and awareness of the SMFPs viewed (Supplementary Table 2). This took approx. 5–10 min for each participant. With regard to appreciation, participants were asked to think about the social media viewing session and indicate in general how much they liked food promotions or food ads on social media (i.e., 5-point Likert scale: ‘Dislike very much’—> ‘Like very much’), and on which of the viewed platforms they considered them to be most appealing. Moreover, they were asked to name types of food promotions or food ads they (dis)liked more than others (i.e., open-ended question). Two measures commonly used to assess level of awareness of stimuli [26] were investigated, i.e. recall and recognition. With regard to recall, participants were asked to name any food promotion or food ad from the viewing session they remembered in particular. To gain insight into the participants’ perceived recognition of SMFPs, they were asked to think about the social media viewing session and indicate how often they thought they would have recognised the examples pointed out by the researcher themselves (i.e., 5-point Likert scale: ‘Never’—> ‘Always’). Participants were always asked why they had certain opinions, and were encouraged to mention examples from the platforms viewed, or to name examples from their social media platforms in general, if none or too few food promotions were encountered during the activity. After the interview, participants were mailed a $30 department store gift card. The study incentive value was increased to $60 in a later phase of recruitment, to generate further participant interest and increase the recruitment rate.

### Data processing and analysis

#### Selection of images

All Zoom screen recordings were securely stored for up to 14 days until both the principal researcher and a second researcher had independently viewed each recording and made screen shots of social media posts (images or video) that contained any branded or unbranded food content. The full screen recording was permanently deleted after this. One social media post could show several food items or brands, and could consist of several videos or photos, e.g., stories within Snapchat or Instagram, and therefore several screen shots were made to keep as much relevant information about the social media post as possible. Food content included images or videos of food brands (i.e., logos or signs), foods (i.e., cartoon or real food), or texts mentioning food (e.g., brand names). The latter included descriptions, hashtags or titles of posts, or posts in text format only, and excluded the comment section of posts, or the names of accounts. A food post was only selected when at least half of the food item or brand (name/logo) was visible. The shortest exposures were included by means of a slow-motion function, as it has been shown that people are able to predict the energy and macronutrient content of food images after exposure of less than 100 ms [27]. Only visual food content was included, and speech-based mentions of foods or brands were excluded from coding. It was expected that some SMFPs may be hidden and unrecognisable. Therefore, to make sure all SMFP exposures were captured, the two researchers compared their image selections, and combined all food-containing images into one set of food content images for each participant. Because the researchers had different backgrounds, i.e. European and Australian, they complemented one another regarding food and brand recognition, as many products or brands from different parts of the world were recognised by at least one of the two researchers.

#### Anonymising the image data

The principal researcher de-identified all retained images by blurring faces and names in the images of all social media posts, except for posts that were sponsored adverts. Next, the researcher deleted the identifiable images, and sent the participant an email with link to the de-identified selection of images from their social media feeds, to show them how they were de-identified and used for analysis. This gave participants an opportunity to withdraw data if preferred. Also, participants were informed that the original Zoom screen recording was deleted from the University’s servers.

#### Coding of images

The two researchers independently viewed a subset (*n* = 150) of de-identified images and coded them, according to a pre-defined coding scheme in Microsoft Excel. This scheme was largely constructed based on other studies in this area [8, 23, 24], and additionally included a variable on the level of food integration, as this was considered relevant in the social media context [14]. The researchers compared the two coded subsets and discussed differences. Based on these discussions, significant changes in the coding structure were applied. For instance, due to time constraints several variables, e.g., tone of voice, message purpose, portion size or amount, were removed from the original dataset. The omitted variables were beyond the scope of the current analysis, and coding them would be rather complex and time consuming. Next, a smaller subset of images (*n* = 70) was coded independently, and only minor issues and differences were discussed and resolved with the research team. The principal researcher continued coding based on the updated coding scheme. Relevant coding variables included the source or content generator, type of content (i.e., paid content, owned content, user-generated content, celebrity-generated content or content embedded in other web content), food classification (i.e., core, mixed, non-core, miscellaneous), food company promoted and level of integration. The coding of food classification was based on the International Network for Food and Obesity/Non-communicable Diseases Research, Monitoring, and Action Support (INFORMAS) food classification system, which is in line with a similar Australian analysis of adolescents’ social media platforms in 2018/2019 [8]. Kelly et al. were contacted to discuss the application of this system to ensure consistent coding. Regarding miscellaneous foods, sub categories were created including mixed dishes, composite foods or drinks, or miscellaneous single foods. The latter mostly concerned unbranded foods of which the exact nutrient composition was unknown, such as cheese, milk or bread. Mixed dishes were categorised as miscellaneous when the ingredient composition was not fully retrievable or when they contained only healthy choices. Supplementary Table 3 shows decisions regarding to composite foods or mixed dishes, which were informed by previous research [8].

#### Data analysis associated with social media posts

Data analyses were conducted using SPSS for Windows version 25 (IBM Corporation). Socio-economic area was calculated based on postal code (low: Q1 and Q2; medium: Q3 and Q4; high: Q5), according to the Socio-Economic Index for Areas (SEIFA) Index of Relative Socio-Economic Disadvantage (IRSD) ranking for Australia, and similar to the method of Gascoyne et al. [17]. Descriptive analyses were conducted to assess the characteristics of all social media food posts, also including separate analyses for branded and unbranded posts.

First, the total number of social media food posts encountered per Zoom interview and per platform were analysed, after which the rates of social media food posts per 10 min were calculated based on the number of social media food posts per participant divided by the number of platforms viewed during the Zoom interview. The data did not meet normality assumptions, based on the Shapiro–Wilk test of normality, and therefore median rates and interquartile range (IQR) were reported. Total numbers and median rates of unbranded and branded food posts were compared using a Mann–Whitney U test.

#### Data analysis associated with the adolescent interviews

Answers to the Likert scale questions were analysed by means of descriptive analyses in SPSS. Answers to the open-ended questions were transcribed based on audio recordings. The qualitative interview was used to compliment the quantitative data and thus it was not the main focus of the analysis. For that reason, and due to the relatively high volume of quantitative data, coding of the interview transcripts was done by one researcher (first author) with input from the research team and pre- and post-analysis discussions with the second author. This author had also viewed all screen recordings, including the social media scrolling activity and participant responses to the interview questions. A directed thematic content analysis was conducted [28], i.e., an initial coding scheme was developed based on the interview guide. Since the main focus of this research was to use the HoE framework to qualitatively explore factors that may determine adolescents’ consumption behaviour as a result of their exposure to food promotions, cognitive (awareness) and affective (appreciation, liking) factors were coded deductively. The coding scheme was further updated based on the interview transcripts. The analysis allowed for new themes to emerge inductively, in order to obtain full insight into adolescents’ perceptions. Participants’ quotes were selected to illustrate their reasoning behind certain ideas or views. Additionally, individual participants’ responses were compared with the number and characteristics of posts they viewed during the social media scrolling activity, to provide further insight into potential links between opinions and actual exposure to SMFPs.

## Results

### Study population characteristics

#### Demographics

In total, 35 adolescents participated (boys: *n* = 18; girls: *n* = 17). The mean age of the sample was 14.4 (1.2) years, and the majority (*n* = 32) resided in medium socio-economic areas (SEA) (Table 1). During the Zoom interview with two participants, a parent was present during part of the Zoom meeting.

**Table 1:** Sociodemographic characteristics of participants (*n* = 35) and their use of social media platforms

Sociodemographic variables	n (%)
**Gender**	
Male	18 (51)
Female	17 (49)
**Age (years)**	
13	11 (31)
14	8 (23)
15	8 (23)
16	8 (23)
**Socio-economic area**^**a**^	
Low	13 (37)
Medium	19 (54)
High	3 (9)
**Social media platform users**^b^	
YouTube	34 (97)
Instagram	33 (94)
Snapchat	27 (77)
TikTok	23 (66)
Pinterest	18 (51)
Facebook	13 (37)
Reddit	6 (17)
Twitch	2 (6)
Yolo	2 (6)
Spotify	1 (3)
Discord	1 (3)
Twitter	1 (3)
WhatsApp	1 (3)

^a^ Classification based on the postal area (Low: Q1 and Q2; Medium: Q3 and Q4; High: Q5) according to the Socio-Economic Index for Areas (SEIFA) Index of Relative Socio-Economic Disadvantage (IRSD) ranking for Australia [29]

^b ^Participants could select multiple responses

#### Social media use

Participants reported using between two (*n* = 2) and six (*n* = 6) social media platforms in total, with an average of 4.6. The three social media platforms used most often among the participants were YouTube (97%), Instagram (94%) and Snapchat (77%). More than half of the participants reported using Instagram and Snapchat several times a day or more (i.e., 54% and 60%, resp.). The female/male ratio for Pinterest and TikTok use was skewed (83% and 65%, respectively).

Participants picked three favourite social media platforms, except for three (*n* = 3) who were active on only two platforms. While TikTok was not in the top three of most favourite platforms, daily TikTok use was relatively long compared to other platforms, i.e., more than half (53%) of TikTok users reported viewing their TikTok feeds for at least 1–2 h a day. All participants (100%) viewed at least one of their favourite social media feeds on their mobile phone, and 66% reported using a computer at home, or a tablet or iPad (34%) when using social media. The participants’ three main purposes for using their favourite social media platforms were to view posts or updates of friends (35%), messaging or talking to friends (33%), or following the news or latest trends (11%). A minority of participants (4%) used their favourite social media platforms to create or share content (Supplementary Table 4).

### Analysis of social media food posts

In total, 100 social media platforms were viewed during the Zoom sessions. The reason for two platforms not being viewed was that the participants indicated they used Snapchat and Discord only for messaging and/or did not prefer to show or use any other social media platform. For two other participants this was also the case, but they both viewed Pinterest instead of Snapchat during the Zoom session. For the same reason, another participant viewed Twitch instead of Discord.

In 1000 min of screen recordings, 1801 posts were identified that contained unbranded (*n* = 1221) or branded (*n* = 580) food content (Table 2). The overall median (interquartile range) rate of food posts viewed on social media accounts was 12.0 (6.3–20) per 10 min, with a median of 7.3 (2.7–11.3) unbranded and 5.3 (3.3–7.7) branded food posts. In 10 min, girls saw significantly more SMFPs than boys (*p* < 0.01), with a median of 19.0 (12.3–25.8) versus 7.3 (3.9–12), respectively. No significant difference in the median rate of food posts per 10 min was observed between participants from different ages and SEAs.

**Table 2:** Characteristics of social media food posts participants were exposed to during the study

Social media posts (*n* = 1801) from 100 screen recordings	Total number of social media food posts, n (%)			Median rate per 10 min (Interquartile range)		
	**Unbranded**	**Branded**	**Total**	**Unbranded**	**Branded**	**Total**
**Total**	**1221 (68)**	**580 (32)**	**1801**	7.3 (2.7–11.3)	5.3 (3.3–7.7)	12.0 (6.3–20)
**Social media platforms**^**a**^						
Instagram (*n* = 26)	321 (56)	253 (44)	574	6.0 (4–11.8)	7.5 (3.8–12.5)	16.0 (8–22.3)
TikTok (*n* = 15)	88 (57)	66 (43)	154	4.0 (4–7)	5.0 (2–6)	9.0 (7–11)
Snapchat (*n* = 22)	220 (72)	85 (28)	305	8.0 (4.8–13)	2.5 (1–5)	11.5 (7–15.8)
YouTube (*n* = 25)	89 (48)	95 (52)	184	3.0 (1–4.5)	3.0 (1–5)	6.0 (2–10.5)
Pinterest (*n* = 7)	498 (87)	72 (13)	570	38.0 (12–71)	12.0 (4–15)	53.0 (19–83)
**Food classification**^**b**^						
Core	222 (94)	15 (6)	237	1.5 (0.7–3)	0 (0–0.3)	1.5 (0.7–3)
Non-core	641 (63)	379 (37)	1020	2.7 (1.3–5.3)	3.3 (2–5.7)	6.0 (3–11)
Only brand logo or name	N/A	91 (100)	91	N/A	0.7 (0.3–1.3)	0.7 (0.3–1.3)
Miscellaneous foods	313 (82)	69 (18)	382	2.0 (1–3)	0.7 (0.3–1)	2.7 (1.3–3.5)
**Food brand promoted**^**c**^						
Food manufacturer/brand	N/A	335 (100)	335	N/A	2.7 (1.7–4.3)	2.7 (1.7–4.3)
Supermarket/retailer	N/A	28 (100)	28	N/A	0 (0–0.5)	0 (0–0.5)
Chain restaurant/café	N/A	168 (100)	168	N/A	1.0 (0.7–2)	1.0 (0.7–2)
Local restaurant/café	N/A	29 (100)	29	N/A	0 (0–0.7)	0 (0–0.7)
Delivery service^d^	N/A	27 (100)	27	N/A	0 (0–0.3)	0 (0–0.3)
**Type of food content, content generator**						
Paid content^e^	62 (25)	191 (75)	253	0.3 (0–1)	1.7 (0.7–2.3)	2.0 (1–3.3)
* Food manufacturer or brand*	*13 (12)*	*96 (88)*	*109*			
* Non-food company/shop*	*39 (83)*	*8 (17)*	*47*			
* Chain restaurant or café*	*0 (0)*	*39 (100)*	*39*			
Owned food content^f^	13 (22)	47 (78)	60	0 (0–0.3)	0.3 (0–1)	0.3 (0–1)
* Food manufacturer or brand*	*2 (12)*	*15 (88)*	*17*			
* Chain restaurant or café*	*0 (0)*	*9 (100)*	*9*			
* Non-food company/shop*	*11 (73)*	*4 (27)*	*15*			
User-generated food content	71 (65)	38 (35)	109	0.3 (0–1)	0 (0–0.5)	0.5 (0–1.3)
* Everyday user (1–1,000 followers)*	*21 (57)*	*16 (43)*	*37*			
* Micro-influencer (1 K-20 K followers)*	*50 (69)*	*22 (31)*	*72*			
Celebrity-generated food content	176 (62)	109 (38)	285	1.7 (0.5–2.7)	0.7 (0.3–1.7)	2.0 (0.7–4)
* Celebrity influencers (*> *300 K followers)*	*115 (60)*	*76 (40)*	*191*			
* Meso-influencers (20 K-100 K followers)*	*30 (60)*	*20 (40)*	*50*			
* Macro-influencers (100 K-300 K followers)*	*31 (70)*	*13 (30)*	*44*			
Food content embedded in entertainment	297 (73)	108 (27)	405	2.3 (1–4)	0.7 (0–1.5)	3.3 (1–5.3)
* Non-food related creative channel*	*192 (69)*	*88 (31)*	*280*			
* Food-related creative channel*	*105 (84)*	*20 (16)*	*125*			
Unknown source	603 (87)	88 (13)	691			
**Level of food integration**^**g**^						
No focus on food/ in background	55 (40)	81 (60)	136	0.5 (0–0.7)	0.3 (0–1)	1.0 (0.7–2.5)
Focus on food/ clearly visible	676 (74)	236 (26)	912	2.0 (0.7–5)	1.5 (1–3.7)	5.0 (2–7.7)
Character handles or holds food	390 (69)	175 (31)	565	2.5 (1–5.3)	1.7 (0.3–2.3)	4.0 (2–8)
Character consumes food	47 (54)	41 (46)	88	0.3 (0–0.7)	0.3 (0–0.5)	0.7 (0.3–1.3)

^a^ Only social media platforms used by > 5 participants are shown, i.e., excluding Facebook (*n* = 2), Twitter (*n* = 1), Twitch (*n* = 1) and Reddit (*n* = 1)

^b^ Based on the International Network for Food and Obesity/Non-Communicable diseases Research, Monitoring, and Action Support (INFORMAS) food classification system

^c^ Only top 5 most occurring shown. Also includes: (outdoor) markets, food or health-related apps/services, catering companies and food trucks

^d^ Includes meal boxes and online restaurant or cafe ordering applications or websites

^e^ Only top 3 most occurring shown. Also includes: supermarket/retail, local restaurants, (outdoor) markets, food-related and non-food related creative channels, user- and celebrity-generated content, delivery services, food or health-related apps/services, and food trucks

^f^ Only top 3 most occurring shown. Also includes: supermarket/retail, (outdoor) markets, user- and celebrity-generated content, delivery services, food or health-related apps/services, catering companies and food trucks

^g^ Excludes promotions in text format or promotions in which no food or brand is shown (e.g., only in title or hashtags of post)

#### Social media platform

Of all nine favourite social media platforms analysed, most food posts were encountered on Instagram (*n* = 574), Pinterest (*n* = 570) and Snapchat (*n* = 305). The majority of food posts on these platforms were unbranded (56%, 87% and 72%, respectively). Pinterest had the highest median rate of exposure to food-containing posts per 10 min (i.e., 53.0), from the seven Pinterest accounts analysed in this study. For Instagram and Snapchat the median rate of exposure to food content per 10 min was much lower, i.e., 16.0 (8–22.3) and 11.5 (7–15.8), respectively, on > 20 accounts viewed in this study. On YouTube, relatively equal median exposure rates of branded and unbranded food posts where viewed (i.e., 3.0 per 10 min), and for TikTok the median rate per 10 min was higher for branded compared to unbranded posts (5.0 versus 4.0, respectively).

#### Food classification

Of all posts, 13% showed exclusively core foods and 57% showed exclusively non-core foods. A minority of posts contained a mix of core and non-core foods (i.e., 4%). The rest of the posts could not be classified as such, as they showed only brand logos (e.g., restaurants where any food can be served) (5%) or they were coded as miscellaneous (21%). Most miscellaneous foods concerned mixed dishes (14%), followed by single foods (5%), mixed drinks (2%) or a combination of those (1%). The median exposure rate of posts with only non-core foods per 10 min was 6.0 (3–11), and the median rate for posts with only core foods was much lower, i.e., 1.5 (0.7–3). Posts showing only non-core foods were mostly promotions embedded in entertaining content (*n* = 225), paid promotions (*n* = 129), or celebrity content (*n* = 121), and they were most frequently from Instagram (*n* = 368), Pinterest (*n* = 299), or Snapchat (*n* = 174). Promotions showing exclusively core foods were mostly unbranded (i.e., 94%). For posts promoting exclusively non-core foods, the number of unbranded and branded foods were more equal (63% versus 37%, respectively).

#### Type of content & source

For 38% of the posts the content generator could not be identified. Many of these posts were shown in pre-view format, when the participant scrolled past but did not click on it (e.g., this happened a lot on Pinterest). Moreover, in social media platforms such as Snapchat an account page could be viewed, but the number of followers was not always visible, making it impossible to categorise the account. The social media food posts for which the source and thus type of content could be determined, the majority was food content embedded in entertainment (36%) or celebrity-generated content (26%). This mostly included food content from non-food related creative channels (25%) and celebrity influencers with > 300 K followers (17%), respectively. Celebrity posts and entertaining content mostly contained unbranded foods (62% and 73%, respectively). Examples of posts that showed food embedded in entertainment include streaming content of football games with brand names on players’ shirts or on the field, ‘satisfying’ video channels where foods were the main subject, and recipe videos. Of all posts of which the source could be identified, owned posts were found the least (i.e., 5%), while the amount of paid posts was higher (i.e., 23%). For both paid and owned posts the majority contained branded foods (75% and 78%, respectively), and most of these were posted by food brands. Unbranded food content shown in paid or sponsored posts were mostly generated by non-food companies that were showing foods or drinks in their posts to market their product or service. An example of this was a kitchenware company, which demonstrated the use of their products by including foods in their posts. Some paid content was posted by sources other than food companies, including influencer paid partnerships (i.e., all levels from everyday influencers to celebrity influencers) (*n* = 8) or even paid ads by creative channels such as recipe blogs or websites (*n* = 6).

#### Food brand promoted

Social media food posts mostly promoted food brands (*n* = 335) or chain restaurants (*n* = 168). This resulted in a median exposure rate of 2.7 (1.7–4.3) for food brands or manufacturers and 1.0 (0.7–2) for chain restaurants per 10 min. Delivery services, supermarkets and local restaurants were promoted the least (i.e., *n* = 27–29), and the median exposure rate per 10 min was negligible. When food companies were being promoted, this was not always by the companies themselves. A majority of the promotions for food brands or manufacturers and chain restaurants of which the source was known were either influencer content or embedded in entertainment (*n* = 161 and *n* = 86, resp.).

#### Level of food integration

In most SMFPs there was a clear focus on the food without a person being present (median rate per 10 min: 5.0 (2–7.7)), or the food was being handled or held with the entire person visible or only their hands (median rate per 10 min: 4.0 (2–8)). Paid content most often showed a clear focus on the food, while entertaining content most often showed characters handling food. A majority of both integration levels included unbranded foods (74% and 69%, respectively). In contrast, the majority of posts with a lower level of food integration, i.e., where the focus was not on the food, was branded (i.e., 60%). Examples of such food promotions were brand logos shown in the background of a football game or on clothes. Hence, the lowest level of integration was most commonly seen in entertaining content. Only 5% of all food posts showed a character consuming the food, and 46% of these were branded foods. Moreover, celebrity-generated content most often showed this highest level of food integration. On Pinterest and Instagram, foods were most often centrally placed in the post (i.e., 46% of all Instagram posts and 79% of all Pinterest posts), while for Snapchat, TikTok and YouTube most posts showed the food being handled (51%, 40%, 35%, respectively). Compared to other platforms, Instagram and YouTube showed the largest amount of posts with food consumption (i.e., 5% of all Instagram posts and 13% of all YouTube posts).

### Appreciation and awareness of social media food promotions

#### Recognition of food content on social media

A minority of participants said they would have ‘Never’ (*n* = 2) or ‘Rarely’ (*n* = 2) noticed the food promotions themselves when they were pointed out to them by the researcher during the scrolling activity. Almost half of 35 participants (*n* = 17) stated to have ‘Sometimes’ noticed the food promotions themselves, 12 participants had ‘Often’ notice them, and only one (*n* = 1) participant had ‘Always’ noticed them.

Fifteen (*n* = 15) participants indicated they were somewhat doubtful whether they were seeing food promotions on social media, in general or during the scrolling activity. The main reason for doubt was related to the covert and subtle way brands are embedded into entertaining themes or aspects of the post, distracting participants from the commercial intent (e.g., recipe videos, Menulog ad with rappers, or Red Bull logos hidden in posts about extreme sports), see Quote 1a, Table 3.

**Table 3:** Participant quotes related to the recognition, recall and appreciation of social media food promotions and marketing

Topic	Quote
**1. Recognition**	
* a. Doubtful about recognition*	*"Sometimes they're going like ‘Oh, buy this brand and bla, bla, bla…’, but sometimes they're just sharing their recipes, and I'm not sure if they're marketing."* (AU07)
* b. No doubt about recognition*	*“I feel like I do recognise a lot of the food ads. I don't really doubt them. But the food ads I get, for example say McDonalds, I don't really go to McDonalds or like it. I guess some of the people I follow follow that. So yes I see and recognise them but I don't go and read through them.”* (AU46)
* c. No attention*	*“I don't really think about it. I might have a quick look at it and scroll past or something.”* (AU55)
**2. Recall**	
* a. Lay-out*	*“It's probably just the fact of different promotional art, and it's usually more colourful and everything.”* (AU60)
* b. Presentation*	*“Because that YouTuber presenting it is extremely famous, and they repeated what they advertised multiple times…”* (AU18)
* c. Type of product/ brand*	*“Probably because I like Guzman y Gomez, and KFC is similar to Maccas. Those are probably the two main ones I go to.”* (AU13)
**3. Appreciation**	
* a. Visual appeal*	*“They make the food look really aesthetic and pretty and make me kind of wanna eat it more.”* (AU86)
* b. Entertainment*	*“I like watching them use the product, like putting it into, like, foods, I guess, using it. So recipes and stuff.”* (AU39)
* c. Temptation*	*“I like more sort of dessert ads, and lolly ads, that sort of stuff, and soft drink ads, they catch my attention more because they're more the type of food that I like.” (AU77)*

Yet, the majority of participants (*n* = 18) indicated that they did not doubt the intent of food promotions or advertisements on social media, for mainly two reasons. Most participants said they did not pay attention to food promotions or advertisements (Quote 1c, Table 3), and some thought they would be able to recognise them (Quote 1b, Table 3).

#### Recall of food promotions

Three (*n* = 3) participants did not recall any particular food promotion or food ad from the scrolling activity. The majority of participants (i.e., *n* = 22) mostly recalled food companies when being asked which food promotions they remembered, while 10 participants mentioned only general foods.

In total, 28 different food brands were mentioned by participants. Food brands that were mentioned more than once by participants include KFC (*n* = 6), Oreos (*n* = 2), Redbull (*n* = 2), KitKat (*n* = 2), Gatorade (*n* = 2). The majority of food brands mentioned (*n* = 21) were non-core or from fast-food restaurants. Other promotions (*n* = 7) were from meal delivery services (i.e., Menulog, Hello Fresh and Uber Eats), and from brands that sell core, non-core or miscellaneous products, including Baker’s Delight, Wrigley’s 5 Gum, Kellogg’s and John West.

When participants mentioned unbranded foods, this mostly included non-core foods such as muffins, cakes, confectionery, ice cream, sausage rolls and donuts. Besides this, participants mentioned fruit smoothies, lasagne and chicken. One participant mentioned corn and two participants mentioned fruit.

Reasons for remembering SMFPs were largely related to the lay-out, presentation and type of product/brand promoted (Quotes 2a-c, Table 3). Being exposed to SMFPs frequently on one or several social media platforms was the most common reason mentioned by participants to remember them better (i.e., *n* = 9). This was followed by the food promotion having a high quality or nice design, i.e., being colourful, shiny and vibrant (*n* = 6), the food in the post looking tasty (*n* = 5), the post standing out (*n* = 4), liking the food brand in the post (*n* = 3), and the food (brand) being prominent (*n* = 3). Other reasons for remembering certain food promotions (i.e., each *n* = 1) include being offered discounts on meal boxes, famous food brands or influencers being shown, simply because the researcher had pointed out the food post to the participant during the session, or it was the most recent food promotion they saw.

#### Appreciation of food promotions on social media

Almost half of participants (*n* = 16) indicated to neither like nor dislike social media posts promoting foods and thus had no clear opinion. Yet, a majority of the participants who had an opinion on them liked food promotions on social media moderately (*n* = 13) or very much (*n* = 4), and only two participants disliked them moderately (*n* = 1) or very much (*n* = 1).

Many participants (*n* = 17) found the food promotions on Instagram most appealing, followed by YouTube (*n* = 7), Snapchat (*n* = 5), Pinterest (*n* = 3), TikTok (*n* = 2). The participants’ reasons for (dis)liking food promotions on social media can be summarised by three main key concepts: visual appeal, entertainment, temptation.

Overall, participants mentioned liking SMFPs with a high visual quality, e.g., that are appealing, aesthetic, vibrant, flashy, eye-catching, refreshing, satisfying, genuine (i.e., not fake), and they liked it when more effort was put into creating them (Quote 3a, Table 3). In line with this, participants did not like plain food promotions that did not stand out. Also, the format of SMFPs was considered relevant by participants, since participants mostly preferred videos rather than photos, although a majority disliked long videos. Participants also disliked pop-up type food ads, which they could not skip and were forced to watch.

Participants repeatedly mentioned liking food promotions that were engaging, entertaining, funny, informative, aligned with their interests, or experimental and out-of-the-box. For example, entertaining content such as videos that show people making recipes were mentioned as appealing by some participants (Quote 3b, Table 3). In line with this, participants did not like food promotions that were repetitive or boring.

Furthermore, participants’ replies suggested they were greatly tempted by the foods shown to them. Thus, they enjoyed seeing foods they would normally like to eat and that looked tasty, including mainly non-core foods such as sweets, see Quote 3c, Table 3. Participants mentioned they did not like promotions showing foods or brands they do not like to eat, including fast food (companies), sweet foods, fruits, vegetables, liquor or beer companies. Reasons for dislike included foods being new to participants, having heard bad things about a company or food, strange food (combinations), or over the top food or brand promotions.

## Discussion

This study aimed to give in-depth and unique insights into the nature and extent of food promotions encountered on social media by Australian adolescents during a real-time social media viewing activity, and adolescents’ appreciation, recall and perceived recognition of social media food promotions (SMFPs).

It was found adolescents are exposed to a median of 12.0 (i.e., 7.3 unbranded and 5.3 branded) SMFPs per 10 min. This is equal to more than 800 food promotions per week, assuming Australian adolescents spend about 1.6 h on social media daily [30]. In their 2018–2019 data, Kelly et al. observed a weighted median of branded food exposures on the web that was almost half the amount of branded SMFPs in this study, i.e., 2.9 per 10 min [8], and Potvin Kent et al. found an even lower median of branded exposures on social media per 10 min, i.e., 2.0 [24] in 2018. This could be an indication that adolescents’ food marketing exposure has largely increased since 2019. Within a few years, social media (use) may have changed significantly, for instance TikTok was not yet as popular in or before 2018–2019, while the current study collected TikTok data from 15 participants. Moreover, the large differences between the results of these three studies may also be attributed to different study populations, i.e., Potvin Kent et al. collected data from Canadian adolescents. Also, differences in methodologies or research settings may have played a role. For instance, while the study by Kelly et al. captured social media data from adolescents in their natural environment (i.e., anywhere they may use social media such as at home or school), participants had control over the data being recorded. In the study by Potvin Kent et al. researchers had more control over the screen recordings, and the study took place in community centres. Additionally, Kelly et al. only included promotions visible for at least 1 s [8], while the current study included even the shortest exposures. Food decisions generally require only limited cognitive processing and can be made in as little as 300 ms [31]. Young adults were even found to be able to estimate energy and macronutrients from food images in less than 100 ms [27], suggesting that measuring the shortest exposures is highly relevant, especially since the nature of social media allows for even shorter and implicit food promotions than traditional media such as TV.

The results of this study indicate the majority of adolescents’ exposure to food promotions on social media concerns non-core foods, in line with previous studies [8, 23, 24]. Similar to this study, Kelly et al. found only 2–3% of branded foods were core foods [8]. This has implications for adolescent health, as exposure to non-core foods on digital media has been associated with a higher intake [4, 16, 17] and better recall of unhealthy foods [32]. In this study, non-core food promotions on social media were indeed remembered more frequently by participants compared to core foods, and they were more often appreciated. The latter may be explained by the source or type of content these non-core food promotions are characterised by, i.e., the majority was embedded in entertaining social media content such as cooking videos, ‘satisfying’ videos, streaming TV content or in vlogs or posts by celebrity influencers with > 20 K followers. The participants in this study generally liked SMFPs when they had the format of (shorter) videos, were entertaining, engaging, informative, experimental, funny, and showed people using the foods, while they disliked more traditional type of food advertisements popping up on their screen. This is in line with other research showing that social media food advertisements are generally seen as more appealing to adolescents than traditional or more obvious food advertising [33]. Of all branded SMFPs in this study of which the source could be retrieved, 72% were user-generated, celebrity-generated or from other entertaining channels. This is a much higher proportion compared to the findings from Kelly et al., who found 59% of all branded food promotions on the web were earned media impressions [8]. Potvin Kent et al. found an even lower percentage of this type of earned marketing exposures, i.e., 49%. This lower percentage of earned marketing in earlier studies may be explained by changes in strategies employed by food companies over time, with a transition towards more marketing through earned content. This suggests food marketers are increasingly relying on entertainment to persuade adolescents, and thus a peripheral or heuristic route to change their brand attitudes, rather than a central or systematic route. According to the Elaboration Likelihood Model (ELM), these two routes have significantly different implications, i.e., the first triggers more affect- and emotion-based mechanisms and unstable brand attitudes while the latter triggers more elaboration or thoughtful consideration and stable brand attitudes [34]. This may be seen in parallel with the cognitive and affective stages underlying consumption behaviour as proposed by HoE models [7], and suggests that food marketers increasingly aim to elicit affective stages through peripheral marketing cues. Social cognitive theories predict that such affect-based marketing messages occur under low involvement conditions, increasing their persuasive effectiveness. Additionally, they propose that repeated exposure to emotional marketing will increase liking of brands though mere-exposure effects and directly prime consumer behaviours [35]. Concerningly, on social media peer-endorsed (earned) marketing blurs the boundaries between online peer activities and advertising and has a greater negative impact than owned or paid media marketing [11, 12], and advertising involving influencers or celebrities are remembered better by adolescents [32].

It was found 38% of all celebrity-generated posts showed branded foods. Yet, marketing disclosure was scarce, with only a small minority being labelled as paid partnerships, and it is likely that many more influencer or user-generated posts without a label were actually paid promotions. Qutteina et al. also found a minority of branded food posts showing a proper marketing disclosure [23]. Some influencers may tag a brand or product or mention them in a hashtag of their post, but this is very subtle and sometimes not even visible to the viewer, e.g., the viewer would need to expand the title. In 2017, the Australian media industry introduced new standards that require food marketing, including food promotions by web-based influencers, to be clearly identifiable from other (advertising) content [36]. However, these rather serve as guidelines and do not pose mandatory restrictions on influencers who are paid to promote food brands. This may have large implications for the adolescent group, as influencer or celebrity brand promotions on social media were found to lead to strong brand effects or increase immediate intake of the promoted foods [37, 38]. According to research by the Advertising Standards Authority (ASA) in the UK, consumers struggle to recognise influencer marketing, and using an ‘ad’ label upfront instead of submerging them in hashtags will increase recognition [39]. Using sponsorship disclosures in social media posts with influencers is particularly essential as posts by brand accounts would automatically elicit more scepticism among consumers [40]. However, previous research does not consistently show that children use disclosures to resist against persuasive messages. For instance, 9–11 year olds who watched an influencer video promoting an unhealthy snack increased their consumption of this unhealthy snack regardless of a disclosure being shown in the video, suggesting the disclosure did not make the children particularly sceptical towards the snack [37]. Moreover, previous work among early (12–14 years) and middle (15–16 years) adolescents showed that activation of knowledge of persuasion in response to a sponsored influencer video depends on the content of the disclosure and on the adolescent phase they are in. It was concluded that early adolescents need more informative disclosures that thoroughly explain about the intent of a persuasive message than middle adolescents, in order for them to use the disclosure to elicit their understanding of influencers’ persuasive intent [41]. However, existing theories suggest that adolescents’ cognitive abilities and understanding or knowledge of persuasive intent are not the only contributors to protecting them from persuasion. The Knowledge Persuasion Model (KPM) emphasises that cognitive ability does not come automatically with age, but as a result of enhanced experience with and exposure to new types of persuasion messages over time [42]. Moreover, according to the more recently proposed Food Marketing Defence Model, besides awareness, understanding and ability, adolescents would need motivation to resist persuasive food messages [35]. Although some participants in the current study seemed interested in knowing more about the source of SMFPs or were somewhat sceptical towards commercial food messages, more than half either did not pay attention to SMFPs or said to not doubt their intent or source. This could suggest that adolescents partly lack the motivation to defend themselves against persuasive food messages. Concerningly, the majority (i.e., 60%) of participants thought they frequently failed to recognise SMFPs, as they said to have noticed them only ‘Sometimes’, ‘Rarely’ of ‘Never’ when they were pointed out to them by the researcher.

Particularly food marketing messages with higher levels of integration into entertainment have been hypothesised to limit children’s capacity to recognise the commercial intent, leading to subconscious persuasion [43], which gradually enhances their implicit memory, attitude and preference of the (non-core) product over time [14, 44]. In this study a small minority of posts were found to have the highest level of food integration, i.e., showing a character’s active consumption of food. This is in line with other research on YouTube and Instagram, although in these studies people were frequently shown holding the food [45, 46]. Indeed, a relatively large number of promotions observed in this study showed a food product being handled or held by a person. Some participants specifically preferred SMFPs with influencers or people showing or handling the foods, i.e., they preferred posts with higher levels of food integration over product placements. This is concerning, as the level of integration between a persuasive commercial message and its context is seen as a key feature of the current and future digital marketing environment [14, 47].

Adolescents were particularly exposed to SMFPs on Instagram, Pinterest and Snapchat. Notably, large numbers of food promotions were observed on Pinterest despite the relatively low number of participants having viewed this platform (*n* = 7). This could be attributed to the format of Pinterest, with many posts being shown in preview format on the screen at once. Also, many posts on Pinterest showed recipes or aesthetic foods rather than branded (sponsored) posts, which explains the large amount of unbranded food posts viewed on this platform. Overall, this study showed a majority of food promotions on social media were unbranded (i.e., 68%). Only a small proportion of promotions for core foods were branded (i.e., 6%), and a relatively larger proportion of non-core food promotions were branded (i.e., 37%). Similarly, previous analyses on YouTube and Instagram found that food promotions were mostly unbranded, and that branded foods promotions mostly concerned non-core foods [45, 46]. This may be reflected in participants’ memory of SMFPs in this study, as most of the food companies participants recalled were fast-food chains or non-core food brands.

This study has some methodological limitations. First of all, participants’ scrolling through their social media feeds may not have been representative to their normal scrolling behaviour for two reasons. Firstly, participants may have altered their social media behaviour because they knew the researcher was viewing their social media feeds with them on Zoom, and secondly, during part of the social media scrolling activity of two participants a parent was present. Second, the coding of composite foods or dishes was done according to the INFORMAS food classification system and partly in consultation with authors of a similar Australian study [8], yet the food classification of these types of foods is often ambiguous and it cannot be ruled out that differences in coding may exist, as their dataset only consisted of branded food posts. As in this study many composite foods and several unbranded single foods such as milk, bread or cheese were difficult to classify based on INFORMAS, a large subset of SMFPs were coded as miscellaneous, and no conclusion could be drawn on their classification. Furthermore, posts showing only the name or logo of a brand selling both core and non-core products were difficult to code as either ‘core’ or ‘non-core’, and were therefore not coded as such. However, this concerned only a small subset of all posts (i.e., 5%). Thirdly, the content generators of many SMFPs could not be identified, i.e., when posts were only viewed in pre-view format or the number of followers could not be retrieved on the account page itself. Fourth, the current study included a convenience sample and only a small subset of the Australian adolescent population, and analysing social media platforms of a larger and more diverse group is potentially needed to obtain data more representative of the Australian adolescent population. Future studies would need to include adolescents from different regions worldwide, as social media usage and adolescent eating practices or health behaviours may differ largely across cultures [48, 49]. Lastly, this study was conducted during the COVID pandemic, which may have led to higher junk food marketing exposure [50].

Yet, the current study is the first study in this area to provide a valuable combination of insights into adolescents’ SMFP exposure and adolescents’ appreciation, recall and perceived recognition of SMFPs. A qualitative analysis of adolescents’ perspectives is highly valuable, as reality is socially constructed and adolescents’ subjective interpretation eventually determines their individual experiences [51]. Regarding the quantitative part of this study, measurements of SMFP exposure were conducted as objectively as possible, with real-time recording of adolescents’ social media feeds instead of content analyses of specific social media brand or influencer accounts, or participants’ self-reported exposure or self-collected social media food marketing data, as applied in previous studies [16, 17, 23, 45, 46, 52]. To date, only two other studies conducted in Canada (i.e. in 2018 or before) and Australia (2018–2019) have measured children's and adolescents’ real-time exposure to food marketing on social media [8, 24]. Particularly, the non-experimental setting of this study and the researcher not being physically present or constantly visible by the participants during the scrolling activity may have resulted in a more natural setting and thus more natural social media behaviour. Also, the current study measured both unbranded and branded food promotions, giving more complete insights into adolescents’ exposure to SMFPs.

Exposure research should be further extended across the globe and in larger population groups, and methodologies to measure adolescents’ exposure to food marketing on social media should be optimised. Namely, while food companies use Artificial Intelligence (AI) to target their engaging and personalised social media marketing to adolescents, AI can also be used for research purposes, i.e., to monitor individuals’ exposure to marketing [53]. Exposure data play an important role in the development and strengthening of food marketing regulations [19]. This study’s findings acknowledge regulations on social media food marketing targeted to children > 12 years should be made stricter to protect this vulnerable group against its implicit and potentially detrimental effects. Yet, unbranded food content is not likely to fall under the scope of any current or future food marketing regulations, while it contributes to adolescents’ exposure to non-core food on social media almost twice as much as branded food content. Therefore, it may have as much impact on adolescents’ dietary behaviours as branded content, especially when they are being shared by peers. Thus, a holistic approach should focus on tackling the effects of both branded and unbranded food content on social media by collaborating with social media influencers and teachers, to educate children about healthy food consumption and digital advertising from a young age. Ideally, this may create a future generation of influencers who encourage normal portion sizes, balanced diets, more realistic body sizes, and who motivate young people to be more sceptical towards digital media sources.

## Conclusions

Based on real-time recordings of adolescents’ favourite social media platforms, this study provides evidence on adolescents’ concerningly high exposure to and appreciation of non-core and entertaining food promotions on social media, and lower exposure to and appreciation of core food promotions and traditional types of food advertising. It is hypothesised that adolescents process these highly engaging SMFPs largely implicitly, and thus without their active awareness prolonged exposure may influence their eating behaviours. Yet, more empirical evidence on adolescents’ exposure to SMFPs and the impact on their food consumption is warranted.

## Supplementary Information

**Additional file 1: Table 1a** Pre-study questions on participants’ socio-demographic characteristics and social media use. **Table 1b** List of most popular social media platforms in Australia (July 2020) and answer categories on how often they are used in the past month. **Table 2** Post-study questions on recognition and appreciation of social media food promotions. **Table 3** Coding of composite food or beverages on social media into core and non-core classifications. **Table 4** Participants’ duration of use, device used to access, and main reason for using their favourite social media platforms.

## Data Availability

The analysed datasets and coding scheme are available from the corresponding author.
