# Supplementary material for: Adolescents’ exposure to and evaluation of food promotions on social media: a multi-method approach
Source: Int J Behav Nutr Phys Act. 2022 Jun 27;19:74. doi: 10.1186/s12966-022-01310-3 (PMC9235222; doi:10.1186/s12966-022-01310-3)
Supplement: Supplementary file 1 — Additional file 1: Table 1a Pre-study questions on participants’ socio-demographic characteristics and social media use. Table 1b List of most popular social media platforms in Australia (July 2020) and answer categories on how often they are used in the past month. Table 2 Post-study questions on recognition and appreciation of social media food promotions. Table 3 Coding of composite food or beverages on social media into core and non-core classifications. Table 4 Participants’ duration of use, device used to access, and main reason for using their favourite social media platforms. [file 12966_2022_1310_MOESM1_ESM.docx]

**Supplementary Tables**

**Table 1a: Pre-study questions on participants’ socio-demographic characteristics and social media use**

| **Demographic questions** | **Answer** |
| --- | --- |
| 1. Are you a boy or a girl? | 0= Boy  1= Girl  2= Other |
| 1. How old are you? | Options: 13, 14, 15, 16 |
| 1. What is your postcode? | Open-ended |
| **Questions about social media use** | **Answer** |
| 1. In the past month, how often have you used the following social media platforms? *(Select one answer per row, by means of an ‘X’)*. If your social media platform(s) is/are not listed in the table, add to the empty rows. | <see Table 1b below> |
| 1. What is your most favourite social media platform? | <choice from list of platforms picked in question 4> |
| 1. Approximately what month of what year did you join this platform?   (*You will be able to find this either on the platform itself or in your email inbox. If you cannot find it, please give us your closest estimation.*) | Date |
| 1. How many other social media users are you connected to on this platform?   (*For example, for Instagram and Twitter this is the number of users you follow, for Snapchat or Facebook this is the number of connections, and for Youtube the number of channels you are subscribed to*) | Number |
| 1. How much time do you spend on this social media platform on a typical day? | 0= not at all  1= 1-30 min  2= 31 min – 1 hour  3= 1-2 h  4= 3 h  5= 4-5 h  6= 6-8 h  7= more than 8 h |
| 1. What device(s) do you use to access this social media platform? *(Several options possible)* | 0= Computer (desktop or laptop) at home  1= Computer (desktop or laptop) at school  2= Computer (desktop or laptop ) at work  3= Mobile phone  4= Tablet (e.g. IPad)  5= Other, please specify:  ………………………………………………………… |
| 1. What do you primarily use this social media account for? *(Choose one option)* | 1= messaging or talking with friends  2= creating or sharing photos/videos  3= viewing posts/updates of friends  4= connecting with (new) people  5= joining groups  6= following the news or latest trends  7= playing games  8= organizing parties/events  9= listening to music  10= other, please specify:  ……………………………………… |
| 1. What is your 2^nd^ favourite social media platform? | < choice from list of platforms picked in question 4> |
| 1. Approximately what month of what year did you join this platform?   (*You will be able to find this either on the platform itself or in your email inbox. If you cannot find it, please give us your closest estimation.*) | Date |
| 1. How many other social media users are you connected to on this platform?   (*For example, for Instagram and Twitter this is the number of users you follow, for Snapchat or Facebook this is the number of connections, and for Youtube the number of channels you are subscribed to*) | Number |
| 1. How much time do you spend on this social media platform on a typical day? | 0= not at all  1= 1-30 min  2= 31 min – 1 hour  3= 1-2 h  4= 3 h  5= 4-5 h  6= 6-8 h  7= more than 8 h |
| 1. What device(s) do you use to access this social media platform? | 0= Computer (desktop or laptop) at home  1= Computer (desktop or laptop) at school  2= Computer (desktop or laptop ) at work  3= Mobile phone  4= Tablet (e.g. IPad)  5= Other, please specify:  ………………………………………………………… |
| 1. What do you primarily use this social media account for? *(Choose one option)* | 1= messaging or talking with friends  2= creating or sharing photos/videos  3= viewing posts/updates of friends  4= connecting with (new) people  5= joining groups  6= following the news or latest trends  7= playing games  8= organizing parties/events  9= listening to music  10= other, please specify:  ……………………………………… |
| 1. What is your 3^rd^ favourite social media platform? | < choice from list of platforms picked in question 4> |
| 1. Approximately what month of what year did you join this platform?   (*You will be able to find this either on the platform itself or in your email inbox. f you cannot find it, please give us your closest estimation.*) | Date |
| 1. How many other social media users are you connected to on this platform?   (*For example, for Instagram and Twitter this is the number of users you follow, for Snapchat or Facebook this is the number of connections, and for Youtube the number of channels you are subscribed to*) | Number |
| 1. How much time do you spend on this social media platform on a typical day? | 0= not at all  1= 1-30 min  2= 31 min – 1 hour  3= 1-2 h  4= 3 h  5= 4-5 h  6= 6-8 h  7= more than 8 h |
| 1. What device(s) do you use to access this social media platform? | 0= Computer (desktop or laptop) at home  1= Computer (desktop or laptop) at school  2= Computer (desktop or laptop ) at work  3= Mobile phone  4= Tablet (e.g. IPad)  5= Other, please specify:  ………………………………………………………… |
| 1. What do you primarily use this social media account for? *(Choose one option)* | 1= messaging or talking with friends  2= creating or sharing photos/videos  3= viewing posts/updates of friends  4= connecting with (new) people  5= joining groups  6= following the news or latest trends  7= playing games  8= organizing parties/events  9= listening to music  10= other, please specify:  ……………………………………… |

**Table 1b. List of most popular social media platforms in Australia (July 2020) and answer categories on how often they are used in the past month [1]**

| **Social media platform** | **Never** | **Once a month** | **Several times a month** | **Once a week** | **Several times a week** | **Once a day** | **Several times a day** | **Once an hour** | **Several times an hour** |
| --- | --- | --- | --- | --- | --- | --- | --- | --- | --- |
| 1. Facebook |  |  |  |  |  |  |  |  |  |
| 2. YouTube |  |  |  |  |  |  |  |  |  |
| 3. Instagram |  |  |  |  |  |  |  |  |  |
| 4. Snapchat |  |  |  |  |  |  |  |  |  |
| 5. Twitter |  |  |  |  |  |  |  |  |  |
| 6. Tumblr |  |  |  |  |  |  |  |  |  |
| 7. TikTok |  |  |  |  |  |  |  |  |  |
| 8. Flickr |  |  |  |  |  |  |  |  |  |
| 9. Pinterest |  |  |  |  |  |  |  |  |  |
| 10. Reddit |  |  |  |  |  |  |  |  |  |
| 11. MySpace |  |  |  |  |  |  |  |  |  |
| 12. RenRen |  |  |  |  |  |  |  |  |  |
| 13. Weibo |  |  |  |  |  |  |  |  |  |
| 14. Foursquare/Swarm |  |  |  |  |  |  |  |  |  |
| 15. Digg |  |  |  |  |  |  |  |  |  |
| 16. Periscope |  |  |  |  |  |  |  |  |  |
| 17. Delicious |  |  |  |  |  |  |  |  |  |
| 18. Other, please specify:.......................... |  |  |  |  |  |  |  |  |  |
| 19. Other, please specify:.......................... |  |  |  |  |  |  |  |  |  |
| 20. Other, please specify:.......................... |  |  |  |  |  |  |  |  |  |

**Table 2. Post-study questions on recognition and appreciation of social media food promotions**

| - - - 1. Think back about the scrolling sessions. When I pointed out a food promotion or food ad to you, how often do you think you had you recognized this yourself? | 1: Never  2: Rarely  3: Sometimes  4: Often  5: Always |
| --- | --- |
| - - - 1. Were you sometimes doubting whether you saw a food promotion or food ad? | Open-ended |
| - - - 1. Can you give an example of a food promotion or food ad you were not sure about? | Open-ended |
| - - - 1. Why do you think you doubted whether it was a food promotion or food ad? | Open-ended |
| - - - 1. To what extent do you think you have become more aware of food promotions or food ads on social media during this social media scrolling session? | 1: Not at all aware  2: Slightly aware  3: Somewhat aware  4: Moderately aware  5: Extremely aware |
| - - - 1. Think about the food promotions and food ads we came across during this social media viewing activity. How much do you like social media posts that promote foods and beverages? | 1: Dislike very much  2: Dislike moderately  3: Neither like nor dislike  4: Like moderately  5: Like very much |
| - - - 1. [In case several social media platforms have been viewed]: On what platform did you find the food promotions or food ads most appealing or attractive? - [If yes]: Why did you find the food promotions or food ads on <mentioned platform> the most appealing or attractive? | Open-ended |
| - - - 1. Are there any food promotions or food ads from the activity that you remember in particular? - [If yes]: Why do you think you remember those in particular? | Open-ended |
| - - - 1. In general, are there any types of food promotions or food ads you like more than others? - [If yes]: why do you like those more than others? | Open-ended |
| - - - 1. In general, are there any types of food promotions or food ads you like less than others? - [If yes]; why do you like those less than others? | Open-ended |

**Table 3. Coding of composite food or beverages on social media into core and non-core classifications**

| **Composite foods or dishes (coded as one entity)** | **Post coded as non-core** | **Post coded as miscellaneous** |
| --- | --- | --- |
| Composite foods/dishes/beverages where not all ingredients can be classified |  | **X** |
| Pizza (rolls) pepperoni | **X** |  |
| Pizza with vegetables and/or other ingredients |  | **X** |
| Dish with fries / hot chips | **X** |  |
| Hamburger or schnitzel dish | **X** |  |
| Quesadilla |  | **X** |
| Salad |  | **X** |
| Sandwich wraps or burritos |  | **X** |
| Sandwiches or toast |  | **X** |
| Baked potato wedges |  | **X** |
| Noodle or pasta-based cooked dish (not instant) |  | **X** |
| Soup (not instant) |  | **X** |
| Mexican dish with nachos/tacos, cheese, tomato, guacamole, ect. |  | **X** |
| Curry or rice-based dish cooked (not instant) |  | **X** |
| Cheesy or creamy dishes (with excessive amounts of cheese or cream) | **X** |  |
| Garlic bread | **X** |  |
| Acai bowl or yoghurt with fruits |  | **X** |
| Overnight oats |  | **X** |
| Pancakes or French toast or waffles | **X** |  |
| Smoothie (exact composition unknown) |  | **X** |
| Coffee or tea drink milk-based (exact composition unknown) |  | **X** |
| Quiche |  | **X** |
| Sushi |  | **X** |
| Fast food- type meal from fast food restaurant | **X** |  |

**Table 4. Participants’ duration of use, device used to access, and main reason for using their favourite social media platforms**

| **Variable** | **n (%)** |
| --- | --- |
| **Active on platform at least 1-2 hours on a typical day, n (% of participants using the platform)**  YouTube (n=24) ^a^  Snapchat (n=24) ^a^  TikTok (n=15)  Instagram (n=26)  Pinterest (n=5)  Discord (n=2)  Facebook (n=2)  Twitter (n=1)  Reddit (n=1) | 11 (46)  10 (42)  8 (53)  7 (27)  1 (20)  1 (50)  0 (0)  0 (0)  0 (0) |
| **Devices used for at least one favourite social media platform, n (% of participants)***  Mobile phone  Computer at home  Tablet or IPad  Smart TV  Computer at school | 35 (100)  23 (66)  12 (34)  2 (6)  1 (3) |
| **Main reasons to use favourite social media platforms, n (% of total number of platforms)**  Viewing posts/updates of friends  Messaging, talking with friends  Following the news or latest trends  Watching other videos or streamers content  Creating/sharing photos or videos  Watching videos: people playing games  Entertainment  For purchasing purposes  For ideas or inspiration  Listening to music | 36 (35)  34 (33)  11 (11)  8 (8)  4 (4)  3 (3)  2 (2)  2 (2)  1 (1)  1 (1) |

*Participants could select multiple responses ^a^ N=1 data is missing

**References**

1. Correll, D. *Social Media Statistics Australia – July 2020*. 2020 3 May 2020]; Available from: <https://www.socialmedianews.com.au/social-media-statistics-australia-june-2019/>.
